# Anemia: Hypodermic Medication

**Published:** 1914-05

**Authors:** 


Anaemia: Hypodermic Medication, M. K. Robbie in *Merck's Archives*:

Iron citrate, 5/6 gr., 0.05 gm.
Sodium arsenate, 1/6 gr., 0.01 gm.
Strychnine sulphate, 1/120 gr., 0.0005 gm.
Sterile distilled water, a sufficiency.

This quantity is put up in a sterile ampoule and given once or twice daily as the case requires. The site of injection is the thigh.

